# Research Review of Thermal Ablation in the Treatment of Papillary Thyroid Carcinoma

**DOI:** 10.3389/fonc.2022.859396

**Published:** 2022-07-01

**Authors:** Di Ou, Chen Chen, Tian Jiang, Dong Xu

**Affiliations:** ^1^ Department of Ultrasound, The Cancer Hospital of the University of Chinese Academy of Sciences (Zhejiang Cancer Hospital), Institute of Basic Medicine and Cancer (IBMC), Chinese Academy of Sciences, Hangzhou, China; ^2^ Key Laboratory of Head & Neck Cancer Translational Research of Zhejiang Province, Hangzhou, China; ^3^ Graduate School, Wannan Medical College, Wuhu, China; ^4^ The Postgraduate Training Base, Wen Zhou Medical University, Hangzhou, China

**Keywords:** thyroid cancer, ablation, papillary thyroid carcinoma, thermal ablation, recurrence

## Abstract

**Background:**

Minimally invasive treatment of thyroid tumors has become increasingly common, but has mainly focused on benign thyroid tumors, whereas thermal ablation of thyroid cancer remains controversial. Clinical studies analyzing the efficacy of thermal ablation of papillary thyroid carcinoma (PTC) have been conducted in several countries to verify its safety. Here, we screened and reviewed recent studies on the efficacy and safety of thermal ablation of PTC as well as psychological assessment, patient prognosis, recurrence, and factors affecting ablation.

**Summary:**

The most significant controversy surrounding ablative treatment of PTC centers on its effectiveness and safety, and >40 studies have been conducted to address this issue. The studies include papillary thyroid microcarcinoma (PTMC) and non-PTMC, single PTC and multiple PTC, and controlled studies of ablative therapy and surgical treatment. In general, ablation techniques can be carefully performed and promoted under certain conditions and with active follow-up of postoperative patients. Ablation is a promising alternative treatment especially in patients who are inoperable.

**Conclusions:**

Clinical studies on PTC ablation have provided new perspectives on local treatment. However, because PTC grows very slowly, it is an indolent tumor; therefore, studies with larger sample sizes and extended post-procedure follow-ups are necessary to confirm the investigators’ hypotheses.

## Introduction

Thyroid cancer is the most common malignant tumor of the head and neck; papillary thyroid carcinoma (PTC) is the most common type, and its incidence is increasing worldwide (1). There are many reasons for the increasing incidence of PTC, among which improved quality of life (QoL), increased health awareness, and the widespread popularity of medical checkups are the main reasons (2–4). A national screening program in South Korea resulted in a 15-fold increase in the incidence of thyroid cancer over a period of 18 years (5). However, the mortality rate of thyroid cancer has decreased because of the slow progression and low risk of most thyroid cancers. These cancers are referred to as low-risk PTCs, and they are characterized by the absence of significant capsule invasion, extra-thyroidal expansion, lymph node metastasis, and distant metastasis (6). Low-risk PTCs are often detected at autopsy or during physical examination. In recent years, local ablation treatment has been applied to low risk PTCs. The ablation methods include radiofrequency ablation (RFA) microwave ablation (MWA), laser ablation (LA), and ethanol injection, and the first three methods are collectively known as thermal ablation. Thermal ablation only damages the tumor while sparing most of the thyroid glands and periglandular tissues, which opens up a new way of thinking about the treatment of thyroid cancer.

## Review

## Thermal Ablation Study of Papillary Thyroid Microcarcinoma

Papillary thyroid microcarcinoma (PTMC) is a thyroid cancer of ≤10 mm in diameter and is usually considered to be a very low risk thyroid cancer (7). PTMC is often considered an inert tumor with a favorable prognosis, and several clinical studies have confirmed the stability of PTMC (8). The American Thyroid Association (ATA), the British Thyroid Association, and the European Society for Medical Oncology recommend regular follow-up for low-risk PTMC, which does not require immediate surgery because of its good prognosis (9–11). One study reported a 20-year survival rate of 99% for PTMC (8). Sugitani et al. (12) analyzed 415 asymptomatic patients with PTMC for up to 22 years of follow-up and showed that only 6% of the nodules increased in size, 91% did not change, and 3% showed shrinkage. Similarly, Professor Ito in Japan reported (13) that 1,235 patients with low risk PTMC treated with observational therapy did not develop distant metastases from thyroid cancer and none of the patients died from PTMC over a period of 1.5–19 years. Although the prevailing treatment remains surgery (14–16), some patients are not eligible for surgery because of systemic disease. Furthermore, surgery may lead to recurrent laryngeal nerve paralysis, hypothyroidism, neck scarring, and lifelong medication dependency (17–20). In addition, surgical treatment has the potential to lead to overtreatment of PTMCs. The 2015 ATA guidelines suggest that patients with low risk PTMCs can also be actively monitored (15); however, some patients are unable to undergo long-term follow-up because of anxiety about the disease, and in these cases, ablation of PTMC is a compromise between surgery and observation. A multicenter PTMC ablation study conducted in China that included 11 centers and 725 patients (21) showed a significant reduction in tumor size at 6 months after ablation compared with that before ablation. On ultrasound, 515 (71.0%) PTMCs disappeared completely. Six (0.8%) patients showed disease progression after ablation, including five (0.7%) patients with new PTMCs and one (0.1%) patient with metastasis in the cervical lymph nodes. Increasing evidence supports that RFA, LA, and MWA can effectively treat PTMC with satisfactory clinical results (**Table 1**).

**Table 1:** Basic information on PTC ablation studies.

N	Study	Institute	year	Study time	country	Types of nodules	Number of patients	Number of nodules	age	Follow-up time	Type of ablation	Average size	hoarseness	Recovery time for hoarseness	Short-term pain and neck discomfort	Other complications	Transient hematoma	recurrence	Lymph node metastasis	Number of nodules completely disappeared	Nodule disappearance rate
1	(33)	Yantai Affiliated Hospital	2014	2010-2013	CHINA	Single nodule	21	21	52.1 ± 13.6	11(3-22) months	MWA	7.3 ± 3.0	4	3month	most	0	0	0	0	5	19%
2	(22)	General Hospital of Chinese PLA	2016	2013-2014	CHINA	Single and Multiple nodules	92	98	44.7 ± 10.7	3-18 months	RFA	5.6 ± 1.8	4	10min-3h	1	0	0	0	0	10	10.20%
3	(61)	Seoul National University Hospital,	2017	2005-2009	KOREA	Single nodule	6	6	N	48.5 ± 12.3 (36–65) months	RFA	9.2 ± 28	0	N	2	0	0	0	0	4	66%
4	(43)	Rui Jin Hospital	2017	2013-2014(Duration of treatment)	CHINA	Single nodule	30	30	N	12-24 months	LA	4.8 ± 1.2	0	N	30	1 patient had a decrease in tsh and an increase in t3t4, and recovered within 2 months	0	0	0	30(Contains 20 cases of scar-like areas)	100%
5	(49)	Beijing Friendship Hospital,	2018	2014-2017	CHINA	Single nodule	46	46	43.63 ± 9.27	42 months	MWA	4.49 ± 1.55	2	13month	26	0	0	0	0	7	15%
6	(34)	China-Japan Union Hospital of Jilin University	2018	2013-2014	CHINA	Single and Multiple nodules	15	21	48.0 ± 8.8	36–48 months	MWA	5.8 ± 2.5	1	10min	8	0	0	0	0	20	95.23%
7	(62)	Rui Jin Hospital	2018	2013-2016	CHINA	Single nodule	64	64	42.5 ± 12.3	25.7 ± 8.2 (12–42)months	LA	4.6 ± 1.5	0	N	0	0	0	2	0	64(Contains 13 cases of scar-like areas)	100.00%
8	(24)	Renji Hospital	2019	2014-2017	CHINA	Single and Multiple nodules	37	38	45.14 ± 12.96	12 months	RFA	6.77 ± 1.92	0	N	0	1 patient’s t4 increased and recovered after 1 month	0	0	0	37	97.37%
9	(45)	Suzhou Hospital	2019	2016-2017	CHINA	Single nodule	37	37	43.9 ± 17.6	16.5 ± 6.9 (12-24) months	LA	5.1 ± 3.4	0	N	34	1 patient coughed; 1tsh increased, t3t4 decreased, and recovered within 3 months	0	0	1	36(Contains 24 cases of scar-like areas)	86.49%
10	(63)	Beijing Friendship Hospital,	2019	2013-2018	CHINA	Single nodule	168	168	47.36 ± 10.75	753 ± 520 (79–1787) days	MWA		6	Transient	0	1 patient with permanent voice impairment	0	2	5	34	20.24%
11	(25)	Soonchunhyang University Seoul Hospital	2019	2008-2017	KOREA	Single and Multiple nodules	133	152	46 ± 12	39 ± 25(6-104)months	RFA	4.3 ± 1.4	1	2month	1	0	1	0	0	139	91.40%
12	(37)	China-Japan Union Hospital of Jilin University	2019	2015-2017	CHINA	Single and Multiple nodules	185	206	42.2 ± 11.7	20.7 ± 8.8 (12–36) months	MWA	5.3 ± 1.91	5	1day-4month	21	0	11	1	0	174	84.50%
13	(64)	Cancer Hospital of the University of Chinese Academy of Sciences	2019	2016-2017	CHINA	Single nodule	107	107	44.08 ± 13.13	12–18 (15.14 ± 3.01) months	Thermal ablation	5.9 ± 1.8	0	N	0	0	0	0	0		
14	(50)	Rui Jin Hospital	2019	2014.1-2014.12	CHINA	Single nodule	36	36	41.5 ± 11.3	49.2 ± 4.5 (30–54) months	LA	4.7 ± 1.4	0	0	0	1 patient had a decrease in tsh and an increase in t3t4, and recovered within 2 months		2	1	36(Contains 2 cases of scar-like areas)	100.00%
15	(26)	University of Ulsan College of Medicine, Asan Medical Center	2020	2019-2020	KOREA	Single and Multiple nodules	74	84	46 ± 12	60-124(72 – 18)months	RFA	4 ± 1.6	1	2month	1	0	2	0	0	84	100%
16	(41)	Dipartimento di Oncologia ed Emato-Oncologia, Università degli Studi di Milano	2020	2018-2020	ITALY	Single nodule	11	11	49.3 ± 8.7	10.2(1.5-12) months	LA+RFA	7.9 ± 1.3	2	N	3	0	0	0	0	N	N
17	(35)	China-Japan Union Hospital of Jilin University	2020	2014-2014	CHINA	Single nodule	41	41	46.10 ± 8.85	>60 months	MWA	2.8-10.0	2	10min, 2m	0	0	0	0	0	40	97.56%
18	(23)	Medical School of Chinese PLA, Beijing	2020	3014-2016	CHINA	Single and Multiple nodules	198	204	42.5 ± 9.5	24-54 months	RFA	6.34 ± 1.8	5	1mon	4	0	0	1	0		almost100%
19	(58)	School of Medicine, Nankai University	2020	2014-2018	CHINA	Single nodule	66	66	41.0 ± 9.2	20.5 ± 7.4 (12–48) months	RFA	13 ± 2	0	N	2	0	0	2	1	38	57.60%
20	(65)	Medical School of Chinese PLA	2020	2016-2018	CHINA	Single and Multiple nodules	202	211	42.79 ± 10.13	24.42 ± 9.15 (3-42) months	RFA	5.35 ± 1.63	0	N	0	0	0	0	0	139	65.88%
21	(36)	Shanghai Tenth People’s Hospital,	2020	2010-2018	CHINA	Single nodule	119	119	48.7	37.2 ± 20.9 (12-101) months	MWA	6.9 ± 1.9	8	2-3month	0	4 patients coughed	1	0	1	89	78.10%
22	(48)	General Hospital of Chinese PLA	2020	2013-2013	CHINA	Single nodule	94	94	45.4 ± 10.8	>60 months	RFA	6.14 ± 2.54	0	N	0	0	0	1	0	N	N
23	(66)	Rui Jin Hospital	2020	N	CHINA	Single nodule	34	34	37.9 ± 10.1	18-30 months	MWA	5.0 ± 1.4	0	N	0	0	2	0	0	32	94.12%
						Single nodule	33	33	41.8 ± 13.4		LA	4.5 ± 1.6	1	3month	0	0	1	0	0	27	81.82%
24	(21)	China-Japan Friendship Hospital	2021	2015-2020	CHINA	Single nodule	725	725	46 ± 11	21 ± 13 (6–60) months	Thermal ablation	6.4 ± 1.8	14	1-6month	0	1 case of cough and 1 case of paroxysmal arrhythmia.	4	5	1	515	71.00%
25	(68)	Nankai University	2021	2014-2019	CHINA	Single nodule	95	95	66 ± 4.4	36.6 ± 16.6 (15–74) months	RFA	6.07 ± 1.96	0	N	1	0	0	1	0	44	46.30%
26	(59)	the First Medical Center of the Chinese PLA General Hospital	2021	2014-2019	CHINA	Single nodule	94	94	43.94	12-36 months	RFA	N	2 cases of complications, the specifics are unknown		0	0	0	4	0	94	100%
27	(42)	The Third Xiangya Hospital	2021	2012-2015	CHINA	Single nodule	105	105	44.1± 12.2	65.4 ± 6.3(60-96) months	LA	6.34 ± 2.62	0	N	27	1 patient had a decrease in tsh and an increase in t3t4, and recovered within 3 months	0	1	2	103	100%
28	(52)	First Medical Center of General Hospital of Chinese PLA	2021	2014-2018	CHINA	Single nodule	115	115	44.9 ± 10.4	26(11-60) months	RFA	6.5 ± 1.9	2	1-3month	115	0	0	1	0	115	100%
29	(69)	China-Japan Friendship Hospital	2021	2014-2020	CHINA	Single nodule	106	106	44.39 ± 11.13	25 ± 11 (9–48) months	MWA	7.7 ± 3.5	6	3-6month	0	0	0	2	2	71	70%
30	(70)	Nankai University	2021	2014-2019	CHINA	Single nodule	91	91	40.7 ± 9.3	36 months	RFA	14 ± 2	0	N	2	0	0	3	1	91	100%
31	(71)	The First Medical Center of Chinese PLA General Hospita	2021	2014-2018	CHINA	Single nodule	12	12	41.0 ± 9.2	24.1 ± 6.9 (13–33) months	RFA	15.25	0	N	0	0	0	0	0	2	16.66%
32	(72)	Medical School of Chinese PLA	2021	2014-2017	CHINA	Single nodule	414	414	43.56 ± 9.79	42.15 ± 11.88 (24–69) months	RFA	5.22 ± 1.59	0	N	16	0	0	10	4	336	88.41%
33	(74)	Chinese PLA General Hospital	2021	2014-2018	CHINA	Single nodule	424	424	44.1 ± 9.5	48.1 months	RFA	5	0	N	0	0	0	10	3	383	90.33%
34	(75)	Chinese PLA General Hospital	2021	2014-2018	CHINA	Bilateral	47	100	43.39 ± 9.26	47.77 ± 11.5424-48)months	RFA	4.81 ± 1.57 (0.20–0.93)	0	N	4	0	0	2	0	92	92%
35	(76)	Chinese PLA General Hospital	2021	2014-2018	CHINA	Single nodule	432	432	43.59 ± 9.68	49.25 ± 12.98(>24)months	RFA	6.03 ± 1.87	0	N	20	0	0	10	5	390	90.28%
						Multiple nodules	55	114	44.09 ± 9.89		RFA	6.29 ± 1.85	0	N	3	0	0	1	1	109	95.61%
36	(77)	Zhejiang University School	2021	2017-2020	CHINA	Single nodule	157	157	45.10 ± 10.25	18-30 months	RFA	5.26 ± 1.74	2	N	0	1 case of transient subclinical addition and subtraction	0	0	0	39	29.30%
37	(27)	The First Affiliated Hospital of Dalian Medical University	2021	2014-2018	CHINA	Single and Multiple nodules	102	109	43 ± 19	60 months	RFA	5. ± 2.9	2	N	0	2 patients had transient subclinical subclinical hypothyroidism; 3 patients had a decrease in tsh and an increase in t3t4, and recovered within 1 week	0	0	2	109	100%
38	(82)	First Medical Center of General Hospital of Chinese PLA	2020	2014-2018	CHINA	Single nodule	112	112	44.9 ± 10.6	13 ~ 60 months	RFA	6.5 ± 1.9	0	N	0	0	0	1	0	112	100%
39	(38)	Affiliated Hospital of Integrated Traditional Chinese and Western Medicine, Nanjing University of Chinese Medicine	2021	N	CHINA	Single nodule	73	73	38.71 ± 11.82	>12 months	MWA	5.8 ± 1.6	1	3month	0	Subclinical addition and subtraction in 2 patients	0	3	3	N	N
40	(60)	The Affiliated Hospital of Qingdao University	2021	2016-2018	CHINA	Single nodule	63	63	43.6 ± 14.2	24 months	MWA	0.45 ± 0.11	Unknown	few days	63	20 patients with hyperthyroidism, including 9 cases of clinical hyperthyroidism and 11 cases of subclinical hyperthyroidism	0	0	0	55	87%

N, This was not reported in the study.

### RFA

RFA is a treatment method that introduces electrodes into the lesion area to generate thermal effects by releasing radiofrequency currents, thereby achieving a high local temperature and inducing solidified necrosis and tissue degeneration. RFA is widely used in the treatment of solid tumors, and it has been applied in PTMC since 2016, when RFA was used to ablate 98 PTMCs in 92 patients in China (22). The results of the study showed a 100% volume reduction rate (VRR) at a follow-up of 18 months, with no tumor recurrence or metastasis during the follow-up period. Ultrasound-guided core needle biopsy (CNB) histopathology confirmed the lack of residual tumor or recurrence. The same group then conducted a clinical study with a larger sample size (23) in which RFA was performed on 204 cases of PTMC. The nodules disappeared completely after 24 months of follow-up, the VRR was 100%, and there were only nine cases of mild complications. In 2019 (24), a retrospective analysis of the safety, effectiveness, and long-term efficacy of RFA treatment was performed including 38 PTMC nodules in 37 patients, and similar results were obtained. In addition to studies in China, Korean scholars (25) followed 133 PTMC patients who refused surgery and had a high-risk for surgery for 12 months after RFA; the results showed that 91.4% (139/152) of the tumors disappeared and all patients were free of recurrence and metastasis. The overall complication rate was 3%. PTMC is considered to be an inert cancer, and although some studies have shown complete ablation of RFA with no recurrence, this does not prove the importance of this intervention in the short term. Studies have thus increased the follow-up period and included a larger sample size. Cho et al. (26) reported complete disappearance rates of 98.8% and 100% at 24 and 60 months after RFA for PTMC, respectively, with no local tumor progression, lymph node metastasis, distant metastasis, delayed complications, surgery-related death, or delayed surgery. A 5-year retrospective study of RFA for PTMC was also conducted in China (27), and all nodes were completely absorbed at the end of follow-up, with only 2 patients developing ipsilateral lateral cervical lymph node metastasis. These studies confirm the effectiveness and safety of RFA for the treatment of PTMC.

### MWA

Compared with RFA, MWA is a relatively new technique that has been widely used for ablation of various benign and malignant tumors, including benign thyroid tumors (28–30). MWA provides a larger ablation area than RFA, which decreases the time necessary to complete treatment and improves tumor inactivation. MWA is thus less susceptible to heat sink effects. The heat sink effect is an important factor leading to local tumor recurrence after RFA (31, 32), which has increased interest in the MWA technique. The first study of 21 patients with PTMC treated with MWA was conducted and reported in China in 2014 (33). The patients were followed-up for 11 months, and no recurrence or metastasis was detected. Eight of the patients underwent ultrasound-guided biopsy, and the pathology results showed no signs of recurrence; four patients recovered in a short period of time after suffering from hoarseness. Teng et al. used low power MWA (34) to treat 15 patients with a total of 21 nodules. After a follow-up period of 3 years, 20 nodules completely resolved without recurrence. However, studies to date have included a small number of cases and a short follow-up time. The long-term effectiveness and safety of MWA were examined in a study that analyzed 41 cases of PTMC (35); after 60 months of follow-up, the nodule reduction rate was 99.37% ± 4.02%, and no tumor progression was detected during the follow-up period. Yue et al. (36) and Teng DK et al. (37) followed 119 and 185 patients for 2 years after MWA and showed complete absorption rates of 93.9% and 84.5%, respectively; in the former study, one patient developed cervical lymph node metastasis at 26 months of follow-up and underwent successful MWA treatment; in the latter study, only minor complications were observed.

Lu et al. (38) evaluated patients using ultrasonography for up to 24 months after treatment and showed a nodule disappearance rate of 100%. This study performed fine needle aspiration biopsy (FNAB) or CNB of the ablated areas at 3 or 6 months after treatment, and the results showed no atypical or malignant follicular cells by pathological histology. The most common pathological features were fibroblast proliferation (87.18%) and chronic inflammation (82.05%), followed by infarction (53.85%).

### LA

Although most of the ablations for low risk PTMC are currently performed by WMA and RFA, another type of thermal ablation, LA, has achieved some efficacy in the treatment of certain systemic tumors. Apini et al. reported (39) a case of a patient with a PTMC of approximately 8 mm in diameter in the thyroid gland that was found on examination due to cirrhotic decompensation combined with renal failure. The patient was treated with LA. FNAB and CNB were performed at 1 and 12 months after PLA, which detected necrotic material and inflammatory cells but no living tumor cells. The nodule was recurrence-free and continued to shrink at 24 months after treatment. Since then, LA has been used for thyroid cancer ablation. Another scholar reported three cases of PTMC treated with LA in 2014 (40). In 2020, an Italian study (41) reported the results of RFA and LA in 11 patients; all patients were free of recurrence and metastasis after surgery; three patients experienced minor postoperative discomfort, which resolved with medication; there were no complications, and patients had a satisfaction score of 10.

A special study on LA was conducted in China (42) including 105 cases of pathologically confirmed solitary PTMC treated with ultrasound-guided LA at Xiangya III Hospital of Central South University. The mean follow-up was >5 years, and the results showed a 100% nodal disappearance rate at 24 months after ablation with no serious complications such as tracheal, esophageal, vascular, or laryngeal nerve injury. Cervical lymph node metastasis occurred in two patients. In 2017, Ruijin Hospital of Shanghai Jiaotong University reported 30 patients treated with LA; at the final follow-up, 10 ablation areas (33.3%) disappeared and 20 ablation areas (66.67%) remained as scar-like lesions, with no post-treatment tumor regrowth detected (43). Institutional studies of cases treated with LA for longer periods were also reported. Kim et al. (44) followed 90 patients for up to 10 years after PTMC ablation and showed a 100% disappearance rate of nodules by 12 months of follow-up. However, after 17–56 months of treatment, five patients (5.5%) developed a new cancerous lesion and one case of lymph node metastasis was found at 2 months after treatment, which was eventually determined to be an undetected cancer site rather than a recurrence. All recurrent patients were treated surgically and remained recurrence-free for 5 years. Ji et al. investigated the effectiveness of ultrasound-guided percutaneous LA for PTMC (45) and achieved similar results. Regarding the pathological features after LA treatment, three cases of thyroid cancer with total thyroidectomy immediately after LA treatment were reported (40), and the pathology showed tissue destruction and charring, complete loss of the entire ablated area and margins of normal tissue surrounding the tumor, and expression of TTF1 and anti-mitochondrial antibodies indicating lack of viability. However, lymph node micrometastases were found in one of the cases. Therefore, the investigators concluded that LA for PTMC should only be used in a subset of designated patients at this stage. In addition, they indicated LA technology may become the first choice for PTMC in the future when precise identification of microlesions in the thyroid and lymph nodes is achieved.

A systematic review of the three methods (RFA/WMA/LA) (46) was performed including 1,187 patients and 1,284 PTMCs, and the results showed that the volume of PTMC was significantly reduced after ablation with all three techniques. MWA was more effective than the other two techniques, but the difference was not statistically significant. There was no difference between the three techniques in terms of complications and recurrence. A meta-analysis also found that LA treatment was less effective for PTMC reduction (47), but the authors concluded that all three techniques are effective and safe for PTMC ablation and can be used as an alternative to surgery in some cases.

### A Comparative Study of Thermal Ablation Versus Surgical Procedures

The main treatment for PTMC remains surgical resection; however, thermal ablation has been shown to benefit patients with PTMC as an alternative to surgery. Several studies have been conducted to compare the two treatment modalities (48–53), thermal ablation and surgery, and the results show that thermal ablation has comparable efficacy to surgery in the treatment of PTMC. A meta-analysis including 339 thermal ablation and 314 surgical patients (54) showed no statistically significant differences in the rates of local tumor recurrence, lymph node metastasis, distant metastasis, and salvage surgery between surgery and ablation. In a long-term follow-up study (27), two patients treated with thermal ablation developed ipsilateral cervical lymph node metastases; however, a study by Myung et al. (55) showed a recurrence rate of 1.4% in patients followed for 5–6 years after surgery. By contrast, ablation does not affect the prognosis of patients treated with or without surgery, and it preserves at least one side of the thyroid gland in most patients. In a related meta-analysis (56), the incidence of temporary laryngeal recurrent nerve injury after thyroidectomy and total thyroidectomy was 10.1 and 8.1%, respectively, and permanent hypocalcemia occurred in 2.8% and 3.3% of patients. In addition, these patients had permanent surgical scarring in the neck, as well as a decrease in QoL because of the need for lifelong medication. RFA treatment can prevent these complications. Another meta-analysis of seven studies including 867 patients (57) showed that thermal ablation is effective for reducing patient complications and decreasing the length of hospital stay, and it is a relatively safe and cost-effective option for the treatment of PTMC.

## Ablation of PTC (Nodule Diameter >1 cm)

Although the use of thermal ablation in the treatment of PTMC is currently in the clinical research stage, advances in technology and the effectiveness and safety of this technique have led scholars to conduct studies on thyroid tumors >1 cm in diameter. In 2020, the Chinese People’s Liberation Army General Hospital (58) performed RFA in 66 patients with T1bN0M0 PTC (all refused or were unsuitable for surgery), and the results of the study showed a technical success rate of 100% with no major complications. There was a significant reduction in tumor volume. At the final follow-up, the tumor VRR was 99.11 ± 2.44% (range 92.62%–100%), and 38 cases (57.6%) showed disappearance of tumors. Puncture results at 3 or 6 months after ablation showed malignant cells in two lesions (3.0%) and cervical lymph node metastasis in one case (1.5%). Cao et al. (53) performed RFA and MWA in patients with T1N0M0 PTC who volunteered for ablation or were not suitable for surgical treatment. During a mean follow-up of 22 months, the nodule disappearance rate, the disease progression, and the complication rates were higher in the T1b group than in the T1a group; however, thermal ablation showed effectiveness and safety for T1N0M0 PTC. Studies comparing the efficacy and safety of RFA treatment and surgical resection in patients with T1bN0M0 PTC belonging to different age groups (59) showed that RFA was more cost effective and associated with a shorter operative time than the surgical group, but there was no significant difference in tumor progression and complication rates. In addition, subgroup analyses of patients older than forty-five years and patients younger than forty-five years showed no significant differences in the incidence of tumor progression and complications. Hospitalization costs were higher in older patients than in younger patients in the surgical group, whereas no difference was observed in the RFA group. Ablation of stage T1b thyroid cancer has not been assessed clinically on a large scale, therefore, it cannot be used as an alternative to surgery; however, it has been a local clinical treatment preference for patients who lack indications for surgery.

## Complications

The main complications of thermal ablation of thyroid cancer are pain, voice changes, bleeding, and changes in thyroid function. Patients have widespread pain after ablation, with a 100% incidence of postoperative pain demonstrated by Zhou (43), Song (52), and Wang (60). Although criteria for assessing postoperative pain are difficult to establish, the duration of pain was short in all studies, and pain relief occurred within 1 week postoperatively. Hoarseness is one of the most common complications of thermal ablation. The heat generated by RFA or MWA during ablation causes transient damage to the recurrent laryngeal nerve, and some patients experience voice changes, with an incidence of hoarseness of 0%–3.57% and a recovery time of approximately 10 min–6 months (21–27, 33–37, 41–45, 48–54, 58, 59, 61–77). One patient with permanent voice change as a complication was reported. In addition, the rich blood supply to the thyroid gland can lead to transient bleeding at the end of the ablation, although this can be relieved with compression. Finally, a small number of patients experience complications such as altered thyroid function (24, 27, 49, 50, 60, 77) and cough (21, 36, 45) after thermal ablation, which normally resolve during the postoperative period.

## Recurrence

Recurrence and metastasis after ablative treatment of PTC are major concerns for researchers and clinicians as well as patients. Thermal ablation is a new medical technology and there are many uncertainties. Studies including small sample sizes have shown that a fraction of patients have no recurrence and metastasis (22, 33, 34, 43, 49, 60–62), whereas another fraction of patients experience lymph node metastasis and tumor recurrence (24–27, 42, 52, 58, 69, 76). However, the small sample size of these studies makes it difficult to draw consistent conclusions. A study of unifocal low-risk PTMC that included a large sample showed an overall incidence of local tumor progression of 3.62% at a follow-up time of 42.15 ± 11.88 months (range, 24–69 months) (72). One patient (0.24%) was diagnosed with residual cancer, 4 (0.97%) had lymph node metastasis, and 10 (2.42%) had recurrent PTMC. Of the 10 patients who recurred, one opted for active surveillance and showed stable volume at the 1-year follow-up. Nine patients received additional RFA treatment. All lesions were successfully treated and disappeared at follow-up in seven cases, and no distant metastases were detected. In addition, 70% of recurrent PTMC in this study occurred in the contralateral glandular lobe, which could suggest that thyroid lobectomy does not prevent recurrence of low-risk PTMC. A controlled study of 884 patients comparing surgery versus ablation (74) showed no significant differences between the surgical and RFA groups in terms of local tumor progression (9/460 [2.0%] vs. 15/424 [3.5%], P = 0.148), LNM (3/460 [0.7%] vs. 4/424 [0.9%], P = 0.914), recurrent PTMC (6/460 [1.3%] vs. 10/424 [2.4%], P = 0.240) and persistent lesions (0/460 [0%] vs. 1/424 [0.2%], P = 0.298). Recurrence was assessed based on relevant imaging examinations at the time of patient follow-up; however, some patients underwent CNB of the ablated central zone, peripheral zone, and surrounding thyroid parenchyma at 3 or 6 months after ablation to assess tumor recurrence (65). The results showed that in 202 patients with low risk PTMC who underwent CNB assessment after ablation, three ablation areas in the peripheral region showed positive biopsy results for CNB. However, early judgment of recurrence could be made when ultrasound images failed to identify CNB but biopsy could. Another study (73) investigated unifocal PTMC and found residual peripheral tumor tissue after CNB puncture, in two patients who subsequently underwent re-RFA.

## Factors Influencing the Effectiveness and Safety of PTC ablation Therapy

### PTC Near the Capsule of the Thyroid Gland

Whether ablation of PTCs located near the thyroid capsule is feasible remains to be determined. The expert consensus on thyroid ablation of the 2019 edition of the Chinese Medical Doctor Association (69) states that the nodule diameter needs to be <5 mm when performing ablation, but the diameter can be 10 mm if the PTC is located near the thyroid capsule. The association of nodules close to the capsule with the risk of metastasis and recurrence remains controversial. A study that included 174 patients (78) showed that the location of nodules within 1.9 mm of the thyroid capsule is associated with increased risk of lymph node metastasis. Another study (79) showed that a shorter distance between the nodule and the thyroid capsule was associated with a higher likelihood of metastasis. In contrast, a study that included 1,922 patients (80) showed that the distance of the tumor from the thyroid capsule was not associated with lymph node metastasis. Therefore, whether the distance between thyroid tumors and the thyroid capsule is related to lymph node metastasis has not been conclusively established, and whether nodes closer to the thyroid capsule should be ablated needs to be further investigated. A clinical study was conducted to answer this question (69). The study included 71 patients with PTC 0–2 mm from the thyroid capsule. The investigators performed PTC ablation and thyroid capsule ablation with a 25 ± 11 month follow-up, and the results showed that all nodules disappeared during the follow-up period, and the incidence of lymph node metastasis and new tumors was 1.9% (2/106). Although the study showed that thermal ablation is safe and feasible for nodes close to the capsule, the safety and efficacy of thermal ablation of nodes close to the capsule needs to be investigated in additional large-sample multicenter studies.

### PTC Located in the Isthmus of the Thyroid Gland

Approximately 39.2% of PTCs are located in the isthmus of the thyroid gland, and there are no definitive guidelines regarding the treatment of PTCs in the isthmus (81). Similarly, the expert consensus on thyroid cancer ablation developed by the Chinese Medical Doctor Association clearly states that cancer located in the thyroid isthmus is a contraindication to ablation (69). The isthmus is a flattened gland that is close to the anterior cervical musculature and the trachea, therefore thermal ablation is not indicated because ablation could damage the surrounding tissues if not performed properly. However, whether PTC in the thyroid isthmus is an absolute contraindication to thermal ablation remains unclear. A clinical study enrolled 112 patients with PTC in the isthmus of the thyroid gland to analyze the effect of ablation treatment (82). The results showed that at 18 months after the procedure, the nodules disappeared at a rate of 100% and were even completely absorbed at 1 month after the procedure, although one patient had a recurrence at 7 months after the procedure. The study demonstrated the effectiveness of the thermal ablation technique in the treatment of isthmic nodules. Next, the same group compared thermal ablation with surgery in the treatment of thyroid isthmus nodules (52). There was no metastasis or recurrence except for one patient in the RFA group who recurred. However, the operative time, bleeding, hospital stay, and treatment cost were higher in the surgical group than in the RFA group, and the THYCA-QOL score was significantly higher in the RFA group than in the surgical group. Although ablative treatment for thyroid isthmus nodules has not been reported extensively, the current study shows that thermal ablation can lead to a significant improvement in the postoperative QoL of patients and is an alternative to surgery for PTC in the isthmus.

### Age

Although there are indicators or models that can definitively predict issues such as the progression of PTC, age is the only independent prognostic factor affecting thyroid cancer-related mortality (83). Many studies have used 45 years as the cut-off point for staging (84–86). The 5-year survival rate of 65-year-old patients with PTMC who were not treated with surgery is 23% (87).. A clinical study analyzed the outcome and safety of PTMC thermal ablation in patients >55 years of age (68). The results showed that all nodules were completely ablated and the VPR at the last follow-up was 99.78 ± 1.54%; there were no serious complications. One patient developed lymph node metastasis and one had a recurrence, and all were treated with a second RFA with satisfactory results. From the perspective of treatment modality, older patients have more systemic underlying diseases and are at a greater risk of complications from general anesthesia during surgical procedures. In contrast, ablation techniques commonly use local infiltration anesthesia, which is associated with fewer complications and is safer. A comparison of the efficacy and safety of RFA and surgical resection in patients with T1bN0M0 PTC >45 years of age (59) showed that the prognosis was similar in both groups; however, the overall cost and complications were lower in the ablation group, indicating that RFA may be an effective and safe alternative to surgery for the treatment of patients with T1bN0M0 PTC. The effect of age on the results of thermal ablation needs to be validated over a longer period of time and in larger studies.

### Chronic Lymphocytic Thyroiditis (CLT)

CLT is an autoimmune disease characterized by extensive infiltration, fibrosis, and atrophy of the thyroid parenchyma. Approximately 33.3% of PTC cases are associated with CLT (88), and that the coexistence of PTC and CLT is strongly associated with prognosis, lymph node metastasis, and distant metastasis and recurrence rates (89). A study assessing the effect of CLT on the efficacy and safety of thermal ablation in PTMC patients (90) showed that the safety and therapeutic outcomes were not different from those of patients undergoing PTMC alone after ablative treatment for more than 20–48 months. The authors concluded that this study provided a basis for studying the mechanisms of immunomodulation induced by necrosis in thyroid cancer.

### Number of PTCs

The current indications for thyroid cancer ablation studies are limited to solitary PTMC. Multiple PTMCs are divided into unilateral glandular lobe with multiple PTMCs and bilateral glandular lobe with at least one nodule, and the incidence of bilateral PTMC is 10%–30% (91). The main treatment for bilateral PTMC is surgery because bilateral PTMC is considered a high risk factor for tumor recurrence (92, 93). However, studies indicate that bilateral lesions are not associated with an increased risk of recurrence (91). Therefore, this issue remains controversial. According to the 8th AJCC/TNM Mortality Risk System and ATA Risk Stratification (14, 94), bilateral PTMC is classified as stage I with a low risk of recurrence. The safety and efficacy of RFA for bilateral PTMC was analyzed in 47 cases (75). The results showed a complete disappearance rate of 92%, but recurrence was observed in two patients. This study demonstrated the safety and efficacy of RFA for the treatment of bilateral PTMC, and the authors concluded that RFA holds promise as an alternative treatment in patients with bilateral PTMC who are not eligible for surgery. In a study by Teng et al. (37), ablation was performed in 18 patients with two or more tumors, but was not reported separately. A recent study included 55 patients with multifocal PTMC and 432 patients with PTMC alone, and a comparative study was performed (76). After 49.25 ± 12.98 months of follow-up, there were no significant differences in VRR, local tumor progression, and recurrence and metastasis rates. The authors concluded that RFA is a promising treatment method after adequate preoperative evaluation.

## Quality of Life After Ablation in Patients With PTC

Due to the specificity of thyroid cancer treatment, the QoL of patients after treatment has become an essential and important part of the treatment process. The SF-36 scale and thyroid cancer-specific health-related QoL (HRQoL) questionnaire were administered to 100 PTMC patients after treatment (95). The results showed that the main risk factors affecting QoL in patients with PTMC after ultrasound-guided RFA were female gender, psychological burden, inattention, and neuromuscular system and pharyngeal/oral symptoms. Therefore, preoperative examinations are necessary to assess related symptoms, and psychological intervention should be provided after RFA to improve the QoL of PTMC patients after treatment. A study comparing QoL after surgery and PTMC (96) included 54 patients in the PTMC group and 34 patients in the surgical group. The patients were scored on HRQoL using the 36-item Health Short Form Questionnaire (SF-36), Thyroid Cancer Specific Quality of Life, and Fear of Progression Short Form Questionnaire, and the results showed that ultrasound-guided PTMC ablation treatment was superior to surgery in terms of HRQoL, indirectly suggesting that the ablation patients had a higher postoperative QoL than the surgical group.

## Conclusions

The treatment options for thermal ablation of PTC are still controversial. A growing number of researchers have demonstrated the safety and efficacy of thermal ablation with longer follow-up periods and larger sample sizes. Finally, it is hoped that thermal ablation technology will truly benefit patients with PTC.

After reviewing so many studies, it seems to me that for T1aN0M0 PTC ablation techniques are well established and can achieve essentially the same efficacy and fewer number of complications as surgery during clinical treatment. Several academics are already focusing their research on PTC of T1bN0M0, and I think this will be a focus of future research.

## Author Contributions

The three authors collected the information together, CC and DO organized and wrote the article, and DX guide the content and writing of the article. DO and CC contributed equally to this work. All authors contributed to the article and approved the submitted version.

## Funding

This study was funded by the National Natural Science Foundation of China (NO.82071946), National Natural Science Foundation of China (NO.81871370), Zhejiang Province Science and Technology Plan of Traditional Chinese Medicine (NO.2020ZB033).

## Conflict of Interest

The authors declare that the research was conducted in the absence of any commercial or financial relationships that could be construed as a potential conflict of interest.

## Publisher’s Note

All claims expressed in this article are solely those of the authors and do not necessarily represent those of their affiliated organizations, or those of the publisher, the editors and the reviewers. Any product that may be evaluated in this article, or claim that may be made by its manufacturer, is not guaranteed or endorsed by the publisher.
